# Macular retinal nerve fiber layer thickness in retinitis pigmentosa patients with and without optic disc drusen

**DOI:** 10.3389/fopht.2024.1476911

**Published:** 2024-12-05

**Authors:** Alvilda H. Steensberg, Lasse Malmqvist, Mette Bertelsen, Line Kessel, Karen Grønskov, Steffen Hamann

**Affiliations:** ^1^ Department of Ophthalmology, Copenhagen University Hospital – Rigshospitalet, Glostrup, Denmark; ^2^ Department of Clinical Genetics, Copenhagen University Hospital - Rigshospitalet, Copenhagen, Denmark; ^3^ Department of Clinical Medicine, University of Copenhagen, Copenhagen, Denmark; ^4^ Neuro-Ophthalmology Department, Rothschild Foundation Hospital, Paris, France

**Keywords:** optic disc drusen (ODD), retinitis pigmentosa, optic nerve head drusen, retinal nerve fiber layer (RNFL), retinal nerve fiber layer thickness (RNFLT)

## Abstract

**Introduction:**

Retinitis pigmentosa (RP) is a group of inherited retinal dystrophies characterized by progressive photoreceptor degeneration. In a recent study, we reported co-existing optic disc drusen (ODD) at 30%, a prevalence 15 times higher than in the general population. The aims of this study were to a) assess if macular retinal nerve fiber layer thickness (RNFLt) was increased in our cohort of RP patients and b) compare RNFLt between RP patients with and without ODD.

**Methods:**

In this *post-hoc* analysis, optical coherence tomography (OCT) scans of patients with RP and healthy controls were manually delineated, and macular RNFLt measurements were obtained. The analyses were conducted both a) for RP patients without ODD compared to controls and b) for RP patients with and without ODD.

**Results:**

OCT scans of 32 patients with RP and 13 healthy controls were included. Macular RNFLt was significantly increased in RP patients compared to healthy controls and in RP patients with ODD compared to RP patients without ODD.

**Discussion:**

Further studies will explore whether increased RNFLt leads to ODD development through dystrophic calcification or, conversely, if ODD in combination with RP-associated retinal ganglion cell damage causes the increased RNFLt through retrograde axoplasmic stasis.

## Introduction

Retinitis pigmentosa (RP) is a group of inherited retinal dystrophies characterized by progressive degeneration of rod and cone photoreceptors (1). This degeneration initiates inflammatory processes that activate glial cells and induce cytokine production (2, 3). Neural remodeling and glial cell proliferation within the retinal nerve fiber layer (RNFL) may contribute to dysfunctional fluid clearance, and consequently, axons may swell (4, 5). However, the role of the distribution of photoreceptor abnormalities and retinal cellular responses in RP pathophysiology is not fully understood. Increased macular RNFL thickness (RNFLt) has been observed in optical coherence tomography (OCT) scans of patients with RP, although the literature is sparse (4, 5).

In a recent study using detailed OCT scanning of the retina and optic nerve head of patients with RP, we found a surprisingly high prevalence of co-existing optic disc drusen (ODD) at 30% (6), normally only prevalent in 2% of the general population (7). The aim of this *post-hoc* analysis was, first, to test whether macular RNFLt was increased in patients with RP compared to a healthy control group and, second, to compare macular RNFLt between RP patients with and without ODD.

## Materials and methods

OCT scans (Spectralis Software V6.16, Heidelberg Engineering, Heidelberg, Germany) of 13 controls and 40 RP patients, obtained following the Optic Disc Drusen Studies (ODDS) Consortium guidelines as previously detailed in our recent RP study (6), were eligible to be included in this *post-hoc* analysis. The inclusion criteria for all participants in the present study were age 18 years or above and good-quality OCT scans, defined as those in which all retinal layers were clearly visible and therefore suitable for delineation. The exclusion criteria for the controls were no ocular pathology. The exclusion criteria for the RP patients were any other ocular diseases that could potentially affect the retina and optic disc. For the analysis of a potential association between ODD and macular RNFLt, RP patients with prelaminar hyperreflective lines but without ODD were also excluded, as these lines may represent a less expressed form or precursor of ODD (8).

**Figure 1:**
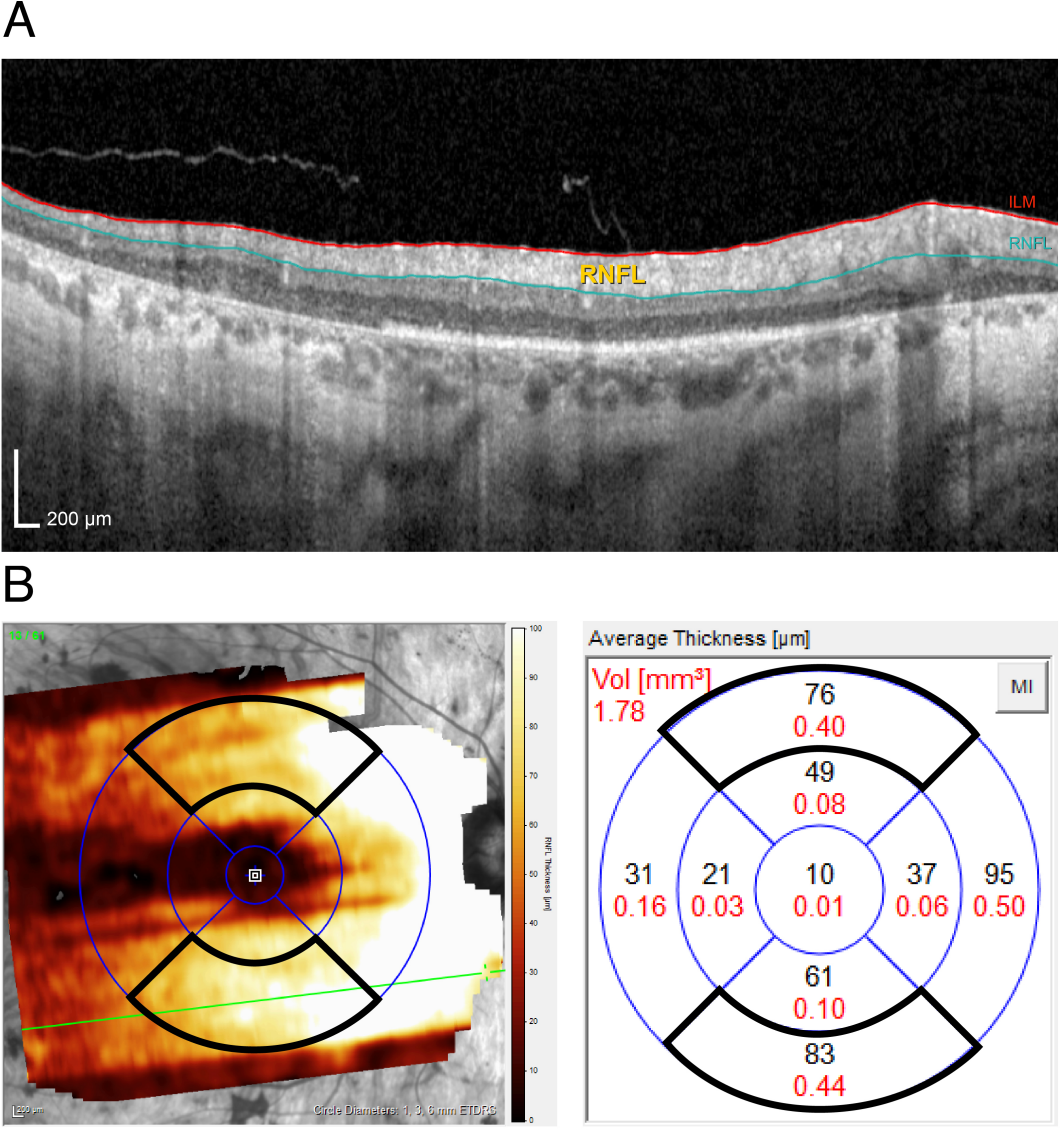
**(A)** Cross-sectional scan showing the retinal nerve fiber layer thickness measured as the distance between the inner limiting membrane (ILM) and the retinal nerve fiber layer (RNFL). **(B)** Macular posterior pole optical coherence tomography scan of the right eye of a 58-year-old patient with retinitis pigmentosa and optic disc drusen. The 3–6-mm ETDRS grid is shown, with the superior and inferior areas framed by a thick black line.

One macular posterior pole scan was analyzed for each individual. For RP patients without ODD and controls, the right eye was selected unless scan quality was insufficient for analysis. For the patients with RP and ODD, the eye with the largest ODD was selected for analysis. Due to retinal degeneration complicating the interpretation of retinal layer separation, the retinal layers were manually delineated. The manual delineation was performed by one author (AHS) and reviewed in a blinded manner by another author (LM)—an ophthalmologist—to ensure accuracy. Based on this delineation, the software provides thickness measurements in areas of a 3-6 mm Early Treatment Diabetic Retinopathy Study (ETDRS) grid, centered on the fovea (**Figure 1**). For this study, the macular RNFLt was calculated as an average of the measurements from the superior and inferior of these areas. The Goldmann visual field area (cm^2^) was used to assess the severity of RP when comparing patients with ODD to those without. Additionally, the disease-causing RP genes were identified to describe the genetic heterogeneity within these groups.

Patients were categorized into two groups: RP patients and healthy controls. RP patients were further categorized based on the presence or absence of ODD. Data distribution was assessed using Q–Q plots and histograms, while the variance of data was evaluated using an F-test. Continuous variables were presented as means and standard deviations (SDs) when normally distributed and as median with interquartile ranges (IQRs) for non-normally distributed data. Group comparisons for normally distributed data were made using Student’s t-test or Welch’s t-test, depending on the variance. For non-normally distributed data, the Mann–Whitney U test was used to compare medians. The statistical level of significance for comparisons was set at p < 0.05.

## Results

For the controls, OCT scans of all 13 individuals were included. Out of the initial 40 eligible RP patients, OCT scans of three were excluded due to poor quality, and scans of five were excluded due to ungradable retinas, resulting in OCT scans of 32 RP patients. For the second analysis, studying RP patients with and without ODD, two additional patients were excluded due to the presence of prelaminar hyperreflective lines but the absence of ODD. This resulted in scans of 12 RP patients with ODD and 18 RP patients without ODD.

Among the RP patients, macular RNFLt ranged from 36.0 to 100.0 µm (mean, 66.9 ± 16.2), while in the controls, macular RNFLt ranged from 30.0 to 48.5 µm (38.4 ± 5.2). The difference between these two groups was significant (p = 1.82·10^−9^, Welch’s t-test), as shown in **Table 1**.

**Table 1:** Characteristics of retinitis pigmentosa patients with and without optic disc drusen and controls.

	Retinitis pigmentosa		Controls	*P* value
Patients, *n* (% male)	32 (59)		13 (39)	
Age, years	47 ± 16		49 ± 12	0.802^a^
Macular RNFL, µm	66.9 ± 16.2		38.4 ± 5.2	<0.001^b^
	With ODD	Without ODD		
Patients, *n* (% male)	12 (50)	18 (61)		
Age, years	40 (38 - 57)	54 (43 - 61)		0.175^a^
Goldmann VF, cm^2^	5.1 (3.4 - 33.6)	8.4 (1.0 - 31.9)		0.440^c^
Macular RNFL, µm	69.2 (61.1 - 79.1)	53.5 (43.0 - 68.0)		0.011^a^

^a^Students t-test, ^b^Welsh t-test, ^c^Mann-Whitney U test. VF, visual field; RNFL, retinal nerve fiber layer; ODD, optic disc drusen.

**Figure 2:**
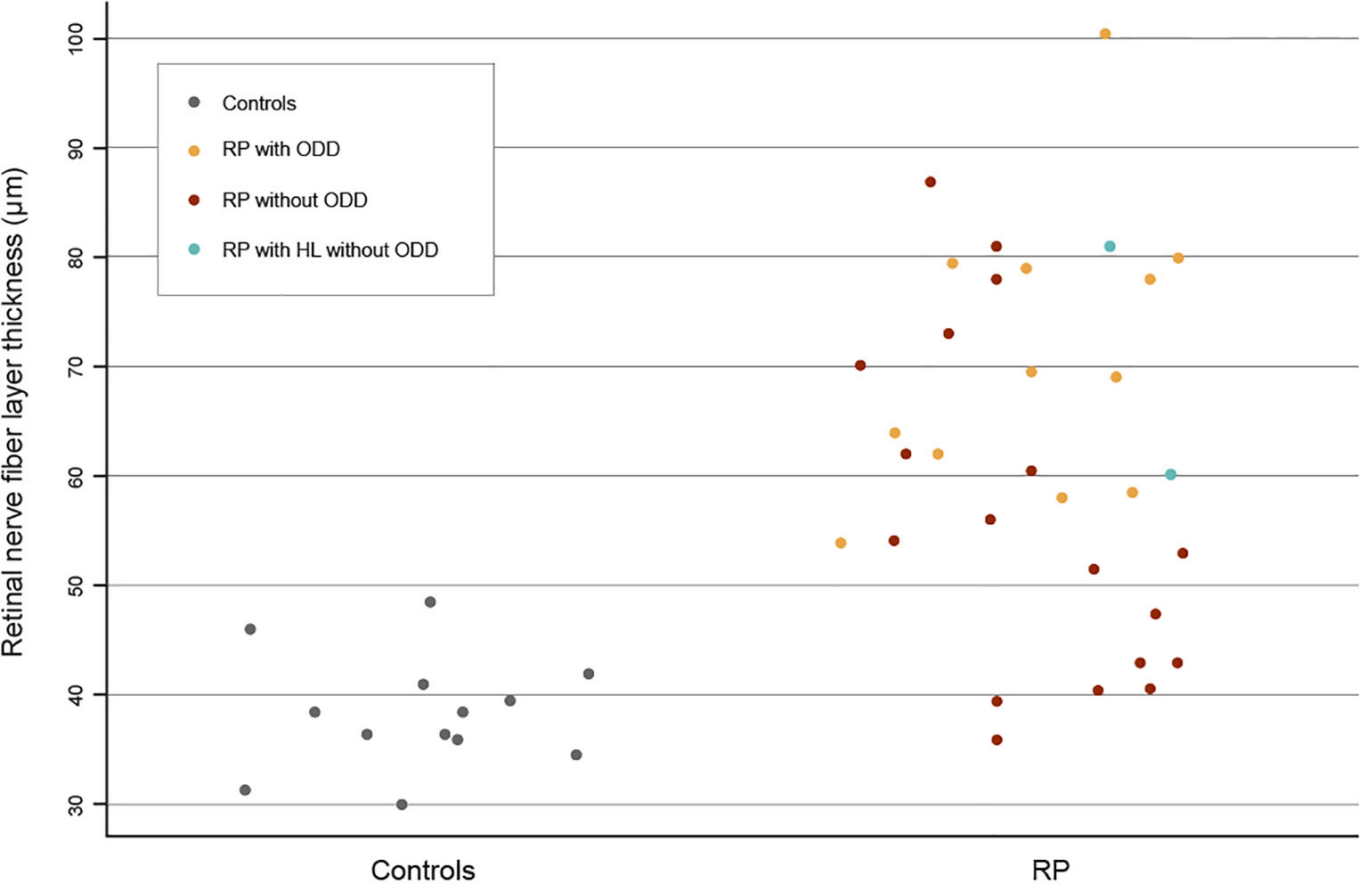
A scatterplot visualizing the data points for the macular retinal nerve fiber layer thickness in controls and retinitis pigmentosa (RP) patients. The RP patients are color-coded by subgroup: RP with optic disc drusen (ODD) in yellow, RP without ODD in brown, and RP with hyperreflective lines (HL) but without ODD in blue.

For the RP patients with ODD, their macular RNFLt ranged from 54.0 to 100.0 µm (median, 69.2 µm; IQR, 61.1–79.1 µm), while in those RP patients without ODD, macular RNFLt ranged from 36.0 to 87.0 µm (median, 53.5 µm; IQR, 42.8–60.8 µm). This difference was also significant (p = 0.011, t-test). All data points are visualized in **Figure 2**.

There were no significant differences between the two groups in the comparison of all RP patients with controls or in the comparison between RP patients with ODD and those without ODD. The Goldmann visual field area of RP patients with ODD was not significantly different from that of RP patients without ODD. The genetic cause of the RP was found in 11 of 12 patients with ODD (nine different genes: RP2, RPGR, USH2A, CNGB1, PCDH15/ABCA4, PRPF31, MYO7A, PDE6B, and KLHL7) and in 13 of 18 without ODD (five different genes: RP2, USH2A, PRPF31, BBS1, and AHI1) (6).

## Discussion

RP patients had an increased macular RNFLt compared to controls, and the measurements for the controls aligned with those reported in a reference database (9). Additionally, we found a significantly increased macular RNFLt among RP patients with ODD compared to those without.

Increased macular RNFLt in patients with RP may result from retinal degeneration, which initiates glial cell activation and proliferation, leading to the production of cytokines and chemokines (2, 3). This potentially initiates axonal swelling as a response to a dysfunctional fluid clearance or altered metabolic demands (10). However, the explanations for the observed phenomenon remain largely speculative.

The increased macular RNFLt in RP patients with ODD can only be speculated. One possibility is that thickening of the macular RNFL in RP compromises axoplasmic transport and causes stasis, potentially damaging retinal ganglion cell axons extending into the optic nerve head and thereby leading to ODD formation via dystrophic calcification processes (11). This would imply that ODD may arise secondarily from ocular changes. However, ODD typically emerges in children, possibly influenced by genetic factors, eye growth, and developmental changes (8). Therefore, the opposite way around is also a possibility, where the presence of ODD in combination with RP-associated retinal ganglion cell damage causes retrograde axoplasmic stasis and swelling of the macular RNFL.

The limitations of this *post-hoc* analysis include its cross-sectional design, which only permits speculation about the nature of the identified associations. Another limitation is the small sample size, which should be considered when interpreting the results and drawing conclusions.

Future, longitudinal studies of the optic nerve head using OCT in RP patients with normal macular RNFLt would be useful to determine whether these patients develop ODD in the subsequent years.

## Data Availability

The data that support the findings of this study are available from the corresponding author, AS, upon reasonable request. Requests to access these datasets should be directed to AS, alvilda.hemmingsen.steensberg.01@regionh.dk.

## References

[1] Hartong DT Berson EL Dryja TP. Retinitis pigmentosa. Lancet. (2006) 368:1795–809. doi: 10.1016/S0140-6736(06)69740-7 17113430

[2] Zhao L Zabel MK Wang X Ma W Shah P Fariss RN. Microglial phagocytosis of living photoreceptors contributes to inherited retinal degeneration. EMBO Mol Med. (2015) 7:1179–97. doi: 10.15252/emmm.201505298 PMC4568951 26139610

[3] Okita A Murakami Y Shimokawa S Funatsu J Fujiwara K Nakatake S. Changes of serum inflammatory molecules and their relationships with visual function in retinitis pigmentosa. Invest Ophthalmol Vis Sci. (2020) 61:30. doi: 10.1167/iovs.61.11.30 PMC7500140 32936303

[4] Yoon CK Yu HG. Ganglion cell-inner plexiform layer and retinal nerve fibre layer changes within the macula in retinitis pigmentosa: a spectral domain optical coherence tomography study. Acta Ophthalmol. (2018) 96:e180–8. doi: 10.1111/aos.2018.96.issue-2 29098796

[5] Hood DC Lin CE Lazow MA Locke KG Zhang X Birch DG. Thickness of receptor and post-receptor retinal layers in patients with retinitis pigmentosa measured with frequency-domain optical coherence tomography. Invest Ophthalmol Vis Sci. (2009) 50:2328–36. doi: 10.1167/iovs.08-2936 PMC2835526 19011017

[6] Steensberg AH Schmidt DC Malmqvist L Kessel L Bertelsen M Grønskov K. Optic disc drusen prevalence in patients with retinitis pigmentosa: A cross-sectional study. J Neuro-Ophthalmology. (2023). doi: 10.1097/WNO.0000000000002038 37976142

[7] Mukriyani H Malmqvist L Subhi Y Hamann S. Prevalence of optic disc drusen: A systematic review, meta-analysis and forecasting study. Acta Ophthalmol. (2023) 102:15–24. doi: 10.1111/aos.15690 37144704

[8] Malmqvist L Li XQ Eckmann CL Skovgaard AM Olsen EM Larsen M. Optic disc drusen in children: the copenhagen child cohort 2000 eye study. J Neuro-Ophthalmology. (2018) 38:140–6. doi: 10.1097/WNO.0000000000000567 28841585

[9] Meyer J Karri R Danesh-Meyer H Drummond K Symons A. A normative database of A-scan data using the Heidelberg Spectralis Spectral Domain Optical Coherence Tomography machine. PLoS One. (2021) 16:e0253720. doi: 10.1371/journal.pone.0253720 34197499 PMC8248651

[10] Ruff A Tezel A Tezel TH. Anatomical and functional correlates of cystic macular edema in retinitis pigmentosa. PLoS One. (2022) 17:e0276629. doi: 10.1371/journal.pone.0276629 36269735 PMC9586413

[11] Bentin JM Heegaard S Jørgensen NR Grahnemo L Hamann S. Optic disc drusen: Dystrophic calcification, a potential target for treatment. Eye (Lond). (2024). doi: 10.1038/s41433-024-03138-6 PMC11306397 38778137

